# The challenge of safe anesthesia in developing countries: defining the problems in a medical center in Cambodia

**DOI:** 10.1186/s12913-020-5068-z

**Published:** 2020-03-12

**Authors:** Kun-ming Tao, Sann Sokha, Hong-bin Yuan

**Affiliations:** 1 Department of Anesthesiology, Eastern Hepatobillary Surgical Hospital, Second Military Medical University, Shanghai, China; 2 Department of Anesthesiology, Preah Ket Mealea Hospital, Phnom Penh, Cambodia; 3 Department of Anesthesiology, Changzheng Hospital, Second Military Medical University, 415 Fengyang Road, Shanghai, 200003 P.R. China

**Keywords:** International standards for a safe practice of anesthesia, Anesthesia safety, Cambodia

## Abstract

**Background:**

The International Standards for a Safe Practice of Anesthesia (ISSPA) were developed on behalf of the World Federation of Societies of Anaesthesiologists and the World Health Organization. It has been recommend as an assessment tool that allows anesthetic providers in developing countries to assess their compliance and needs. This study was performed to describe the anesthesia service in one main public hospital during an 8-month medical mission in Cambodia and evaluate its anesthetic safety issues according to the ISSPA.

**Methods:**

We conduct a retrospective study involving 1953 patients at the Preah Ket Mealea hospital. Patient demographics, anesthetic techniques, and complications were reviewed according to the registers of the anesthetic services and questionnaires. The inadequacies in personnel, facilities, equipment, medications, and conduct of anesthesia drugs were recorded using a checklist based on the ISSPA.

**Results:**

A total of 1792 patients received general and regional anesthesia in the operating room, while 161 patients receiving sedation for gastroscopy. The patients’ mean age was 45.0 ± 16.6 years (range, 17–87 years). The three most common surgical procedures were abdominal (52.0%; confidence interval [CI], 49.3–54.7), orthopedic (27.6%; CI, 25.2–29.9), and urological surgery (14.7%; CI, 12.8–16.6). General anesthesia, spinal anesthesia, and brachial plexus block were performed in 54.3% (CI, 51.7–56.8), 28.2% (CI, 25.9–30.5), and 9.4% (CI, 7.9–10.9) of patients, respectively. One death occurred. Twenty-six items related to professional aspects, monitoring, and conduct of anesthesia did not meet the ISSPA-recommended standards. A lack of commonly used drugs and monitoring equipment was noted, posing major threats to the safety of anesthesia practice, especially in emergency situations.

**Conclusions:**

This study adds to the scarce literature on anesthesia practice in low- and middle-income countries such as Cambodia. Future medical assistance should help to strengthen these countries’ inadequacies, allowing for the adoption of international standards for the safe practice of anesthesia.

## Background

Many developing countries, especially countries with longstanding conflicts such as Cambodia, have a critical shortage of healthcare workers and medical resources [1]. As an important part of medical services, anesthesia services are particularly susceptible to the level of socioeconomic development [2]. In low-income countries, anesthesia services are often provided by unqualified personnel, who are considered a low priority and lack the voice to request access to resources [3].

The quality of anesthesia services is highly correlated with perioperative mortality and morbidity [4]. Identification of the basic problems and resulting demands in anesthesia service is important to better ensure the safety and efficacy of surgical treatments in the developing world. Such data are essential to guide the efforts of governmental and nongovernmental organizations to improve health care delivery in these countries [5]. Unfortunately, with the exception of anecdotal reports and regional evaluations published 20 years ago, few recent studies have focused on the status of anesthesia service in Cambodia [6, 7].

To provide guidance and assistance in maintaining and improving the quality and safety of anesthesia care worldwide, the International Standards for a Safe Practice of Anesthesia (ISSPA) was developed on behalf of the World Federation of Societies of Anaesthesiologists and the World Health Organization [8]. The ISSPA was first published in 1992 and most recently amended in 2018. The ISSPA covers essential areas for anesthesia safety, including professional aspects, facilities and equipment, medications, monitoring, and the conduct of anesthesia [8]. The ISSPA has been recommend as an assessment tool that allows anesthetic departments, institutions, or countries to assess their compliance and needs [8, 9]. In the present study, we described the anesthetic management in one of the largest teaching institutions in Phnom Phen, Cambodia for the first time and evaluated its anesthetic safety issues according to the ISSPA.

## Methods

Ethical approval was obtained from the local ethics committee of Preah Ket Mealea Hospital. Because this was an observational quality improvement study, the need for individual written consent was waived by the hospital ethics committee. The article adheres to the applicable Enhancing the QUAlity and Transparency Of health Research (EQUATOR) guidelines (Strengthening the Reporting of Observational Studies in Epidemiology [STROBE] statement).

The Preah Ket Mealea Hospital is the main public teaching referral center in Phnom Phen, Cambodia. Procedures such as major brain and spine surgeries are routinely performed here [10, 11]. We retrospectively reviewed the anesthetic records of all patients who had undergone operations in the hospital from 1 February to 30 September 2018 either directly or indirectly under the help of a local anesthetist. The patients were requested to provide written consent for surgery and anesthesia. Patients who underwent local anesthesia performed by surgeons were excluded from the analysis. Data were obtained from the registers of the anesthetic services. A standardized form was designed to collect relevant information: age, American Society of Anesthesiologists classification, diagnosis, type of operation, and anesthetic technique. In addition, one questionnaire on the incidence of complications (defined in additional file 1) [12] and another questionnaire on the availability of medications and equipment (based on the ISSPA) were designed to compensate for data that were lacking from the recording charts [13]. At the end of the questionnaires, anesthetists of the department were asked to provide free-text comments on how to improve the safety of anesthesia services.

Two investigators checked the anesthesia personnel, monitoring, and delivery technique in the department according to the ISSPA [8]. The ISSPA includes three levels of standards: highly recommended, recommended, and suggested standards. We checked all levels of standards in this study to fully demonstrate the current situation of anesthesia service. Two investigators (K.M. Tao and S. Sokha) independently filled out the checklists through their observation and work experience, and disagreements were resolved through discussion with a third reviewer (H.B. Yuan). Items that did not meet the standard were recorded for further analyses.

The SPSS 12.0.1 software program (SPSS Inc., Chicago, IL, USA) was used for statistical analysis. Descriptive statistical methods were applied to present the results of single sections and questions. An overall Kappa coefficient was calculated as a measure of inter-rater reliability between two investigators’ ratings on the ISSPA checklist. *P* values of < 0.05 were considered significant.

## Results

### Economic, health care, and anesthesia workforce status in Cambodia

The financial resources allocated to health care are meager in Cambodia (see in additional file 1) [14–16]. Per capita spending on healthcare is 18 times greater in the United Kingdom than in Cambodia. Although the medical education model in Cambodia is identical to the Western model, the number of physician anesthesia providers per 100,000 population is only one-sixth that in the United Kingdom. Additionally, the proportion of anesthesiologists with qualified training experience is even lower.

### Epidemiology of anesthesia service

The records of 1953 patients were included in the study, with 1792 (91.8%) receiving general and regional anesthesia in the operating room and 161 (8.2%) receiving sedation for gastrointestinal endoscopy in the outpatient operating room. The male: female sex ratio was 1.53:1.00, and the mean age was 45.0 ± 16.6 years (range, 17–87 years).

All the patients undergoing surgery were preoperatively assessed by a physician anesthetist. Ninety percent of patients had an American Society of Anesthesiologists grade of 1 or 2. The baseline investigations included an electrocardiogram (ECG) and blood tests for a complete blood count, blood group, coagulation function, electrolyte levels, and liver and kidney function.

The types of surgery performed are presented in Table 1. The three most common procedures were appendectomy, fracture reduction and internal fixation, and cholecystectomy. Emergency surgery accounted for approximately 37.3% of the procedures, and most were performed in patients with trauma or general peritonitis.

**Table 1:** Patients’ baseline characteristics and surgical procedures

Observations (Total *n* = 1953)	n or n (%, CI)
Age^a^	43 (32–57)
Age < 60	1545 (79.1%, 77.0–81.2)
Age ≥ 60	408 (20.9%, 18.8–23.0)
Gender	
Male	1180 (60.4%, 57.9–62.9)
Female	773 (39.6%, 37.1–42.1)
Emergency^b^	728 (37.3%, 34.7–39.9)
ASA grade	
I	1061 (54.3%, 51.7–56.8)
II	711 (36.4%, 33.9–38.9)
III	150 (7.7%, 6.4–8.9)
IV	31 (1.6%, 1.4–1.9)
Types of surgery	
Abdominal surgery	931 (52.0%; CI, 49.3–54.7)
Orthopedic surgery	495 (27.6%; CI, 25.2–29.9)
Urological surgery	263 (14.7%; CI, 12.8–16.6)
Brain surgery	48 (2.7%; CI, 1.8–3.5)
Other	56 (3.1%; CI, 2.2–4.0)
Five most common surgical interventions	1258 (70.2%; CI, 67.8–72.7)
Appendectomy	414
Open reduction and internal fixation surgery	406
Cholecystectomy	175
Urological endoscopic surgery	159
Exploratory laparotomy	104

Abbreviations: *ASA* American Society of Anesthesiologists, *CI* confidence interval

^a^Data are presented as median values and IQR

^b^Emergency refer to the patients received emergency surgeries in the operating room

### Anesthetic techniques

After the patient entered the operating room, the anesthesiologist was responsible for the peripheral venous puncture. Pulse oximetry and noninvasive blood pressure measurement were performed for intraoperative monitoring in all patients. ECG monitoring was rarely used during surgery. Capnography, measurement of body temperature, and monitoring of neuromuscular function were not used in any patients because of a lack of equipment.

General anesthesia was performed in 54.3% of the patients. The combination of diazepam and propofol was used for anesthesia induction, isoflurane was used for maintenance, succinylcholine and vecuronium were used for muscle relaxation, and fentanyl was used for analgesia. Compressed air and oxygen were supplied by cylinders. For all patients undergoing general anesthesia, tracheal intubation and mechanical ventilation were applied with an Aestiva/57900 anesthesia machine (Datex-Ohmeda Inc., Madison, WI, USA). The regional anesthesia techniques performed were mainly spinal and brachial plexus blocks. Epidural anesthesia was rarely performed because of the lack of needles and supplies. At the end of surgery, all patients who underwent regional anesthesia were immediately transferred to the surgical wards, and patients who underwent general anesthesia were observed in a recovery ward. Table 2 lists the five most frequent complications as described by the anesthetists surveyed.

**Table 2:** Details of anesthesia practice

Qualification of individual performing anesthesia	n or n (%, CI)
Physician (specialist) anaesthetist	4
Non-specialist physician anaesthetist (general physician background)^a^	10
Nurse anaesthetist	1
Types of anesthesia	
General anesthesia	1059 (54.3%; CI, 51.7–56.8)
Spinal anesthesia	550 (28.2%; CI, 25.9–30.5)
Brachial plexus block	183 (9.4%; CI, 7.9–10.9)
Sedation for gastrointestinal endoscopy	161 (8.2%; CI, 6.8–9.6)
Medications and fluids commonly used	
Intravenous hypnotic agent	Diazepam,Propofol
Volatile anesthetic agent	Isoflurane
Analgesic agent	Fentanyl
Muscle relaxant agent	Succinylcholine Vecuronium bromide
Local anesthetic agent	Lidocaine,Bupivacaine
Intravenous fluids agent	Crystalloids
Five most frequent perioperative anesthesia complications^b^	
Hypoxia	14
Hypotension and Hypertension	12
Arrhythmia	12
Shivering	10
Anaphylaxis	8

Abbreviations: *CI* confidence interval

^a^A graduate of a medical school who has not completed a specialist training program in anesthesia but has undergone some anesthesia training; ^b^Complications suggested by the anesthesia providers form the questionnaire, data are presented as the number of staff suggested, and the total number of staff surveyed is 15

### Anesthesia-related death

One death was recorded during the study period. A 50-year-old man undergoing surgery for lumbar disc herniation developed sudden cardiac arrest during anesthesia induction. The patient had no preexisting comorbidities. Preoperative monitoring and blood analysis excluded heart disease, hypovolemia (normal heart rate, blood pressure, and pulse oximetry plethysmographic waveform), acute anemia, and electrolyte disturbance as possible causes of cardiac arrest. No ECG or end-tidal carbon dioxide monitoring were being performed when the cardiac arrest occurred. Decreased oxygen saturation and hypotension were first detected after propofol bolus injection. No signs of cutaneous rash or edema were present. Immediately after the cardiac arrest, ECG monitoring, chest compressions, tracheal intubation, and mechanical ventilation were performed. Ventilation difficulty was detected with a rise in the peak airway pressure after intubation. Adrenalin and sodium bicarbonate were then administered via a peripheral vein during cardiopulmonary resuscitation. Return of spontaneous circulation was achieved after 30 min of resuscitation, and the patient was transferred while still intubated and ventilated to the intensive care unit with the support of vasoactive drugs. However, after 3 days of coma (Glasgow coma scale score of 3 on day 3), the family discontinued treatment and the patient died of circulatory failure after 7 days.

### Compliance with ISPPA

According to the ISSPA-recommend checklists, items that did not meet the standards are listed in Tables 4 to 8. The overall inter-rater Kappa coefficient was 0.75, indicating substantial agreement between the two raters.

#### Professional aspects

With respect to professional aspects, the main problem was the lack of available time, facilities, and financial support for professional training of all anesthesia providers. Additionally, no incident-reporting system with case analysis for anesthesia quality control had been established. Because of the nationwide shortage of anesthesiologists, physicians often need to provide anesthesia services in out-of-hospital clinics; thus, the physicians often practice with undue fatigue (Table 3).

**Table 3:** Compliance with ISSPA on professional aspects

Item	Standards	Compliance
**Professional Status**	Anesthesia be provided, led, or overseen by an anesthesiologist	AM
	Local and national standards should be consistent with the ISSPA	PM
**Professional Training**	Formal training in a nationally accredited (postgraduate) education program and documentation of training	PM
**Number of Anesthesia Providers**	The number of anesthesiologists must be adequate to ensure effective leadership of anesthesia services and delivery of care.	AM
**Professional Organizations**	Anesthesia providers should form appropriate organizations at local, regional, and national levels for the setting of standards of practice, supervision of training, and continuing education with appropriate certification and accreditation	PM
**Quality Assurance**	An anonymous incident-reporting system with case analysis resulting in recommendations for alterations in practice	NM
**Workload**	A sufficient number of trained anesthesia providers should be available so that individuals may practice to a high standard without undue fatigue or physical demands	PM
	Time should be allocated for education, professional development, administration, research, and teaching	NM

Abbreviations: *AM* always met, *PM* partially met, *NM* never met

#### Equipment, medications, and monitoring

With respect to equipment and medications, the questionnaire results reflected the lack of supplies and equipment for ECG monitoring, defibrillation, end-tidal carbon dioxide measurement, body temperature measurement, and neuromuscular monitoring (Table 4). This severely limits the monitoring items that can be carried out during and after surgery (Table 5). This was coupled with a lack of commonly used anti-arrhythmia and cardiovascular active drugs (Table 6), making it difficult to handle emergencies such as difficult airways, arrhythmia, and allergic reactions.

**Table 4:** Availability of equipment according to ISSPA

Location	Always available	Sometimes available	Never available
**Preoperative area**	Dedicated space for preoperative assessment		
**Operating room**	Adequate lighting Tilting operating table Supply of oxygen (oxygen cylinders) Oropharyngeal airways Facemasks Appropriate sized laryngoscope and laryngoscope blades for adult patients Appropriate sized endotracheal tubes for adult patients Suction device and catheters Adult self-inflating bags Equipment for IV infusions and injection of medications for adult patients Equipment for spinal anesthesia or regional blocks Sterile gloves Stethoscope Pulse oximeter Electrocardiogram Noninvasive blood pressure monitor with appropriate sized cuffs for adult patients Work surface and storage for equipment and medications System for delivering inhalational anesthesia (plenum) Examination (nonsterile) gloves	Intubation aids (eg, Magill forceps, bougie, stylet) Automated ventilator with disconnect alarm Adult supraglottic airways Infusion pumps Intra-arterial blood pressure monitor Temperature monitor (intermittent) Warming blanket	Access to a defibrillator Carbon dioxide detector IV pressure infusor bag Device for warming IV fluids, blood Continuous waveform capnography Peripheral neuromuscular transmission monitor (nerve stimulator) Inhalational anesthetic concentration monitor Temperature monitor (continuous electronic) Intensive care ventilator
**Postanesthesia recovery area**	Adequate lighting Supply of oxygen (cylinders) Suction device and suction catheters Facemasks Electrocardiography Pulse oximeter Noninvasive blood pressure monitor with appropriate sized cuffs for adult patients Dedicated space for recovering patients Examination gloves (nonsterile)	Adult self-inflating bags Temperature monitor (intermittent)	Access to a defibrillator

**Table 5:** Compliance with ISSPA on intraoperative and postoperative monitoring

Items	Always Met	Sometimes Met	Never Met
**Intraoperative Monitoring**	Audible signals and alarms at all times Continuous use of pulse oximetry Intermittent noninvasive blood pressure monitoring Continuous use of an electrocardiogram Continuous measurement of inspired and expired gas volumes Urine output monitoring (in appropriate cases)	Clinical observation by an appropriately trained anesthesia provider Inspired oxygen concentration monitor Disconnect alarm (when mechanical ventilator used) Continuous measurement and display of arterial blood pressure (in appropriate cases)	Carbon dioxide detector for patients undergoing intubation Device to prevent delivery of a hypoxic gas mixture Intermittent temperature monitoring Peripheral neuromuscular transmission monitor (when muscle relaxants used) Continuous waveform capnographya for patients undergoing general anesthesia and deep sedation Continuous measurement of inspired and expired inhalational anesthetic concentrations Continuous electronic temperature monitoring (in appropriate cases) Processed EEG in appropriate cases
**Postoperative Monitoring**	Continuous use of pulse oximetry Intermittent noninvasive blood pressure monitoring Urine output monitoring (in appropriate cases)	Clinical observation: Tissue oxygenation and perfusion Respiratory rate and quality Pulse rate and quality	Assessment of pain score using age appropriate scale Intermittent temperature monitoring

**Table 6:** Availability of medications according to ISSPA

Items	Always available	Sometimes available	Never available
**Intraoperative medications**	Ketamine Diazepam Fentanyl Local anesthetic (Lidocaine, Bupivacaine) Propofol Thiopental Isoflurane Succinylcholine Vecuronium Neostigmine	Morphine Sevoflurane Rocuronium Midazolam	Cisatracurium Pancuronium Atracurium
**IV fluids**	Saline	Mannitol Ringer’s lactate	Plasmalyte
**Resuscitative medications**	Oxygen Epinephrine Atropine Ephedrine	Hydrocortisone Norepinephrine Dopamine Amiodarone	Dextrose Metaraminol Phenylephrine
**Postoperative medications**	Acetaminophen (paracetamol) Tramadol	Morphine Appropriate nonsteroidal anti-inflammatory medicine (eg, ibuprofen)	Gabapentin Oxycodone
**Other medications**	Furosemide Nitroglycerine Hydralazine	Magnesium Calcium chloride Heparin	Salbutamol Hydralazine

#### Conduct of anesthesia

With respect to the conduct of anesthesia, no safety checklist such as the World Health Organization safe surgery checklist was utilized during the whole process of care. When responsibility for care is transferred from one anesthesia provider to another, the process of handing over patient information is arbitrary. Still, postoperative administration of opioids and other analgesics depends mainly on the doctor’s habits rather than on assessment and certain analgesic modalities (Table 7).

**Table 7:** Compliance with ISSPA on conduct of anesthesia

Item	Standards	Compliance
**Personnel**	One anesthesia provider should be dedicated to each patient and be present in the anesthetizing location throughout each anesthetic.	AM
	A trained assistant (operating room nurse or technician) should be available to assist the anesthesia provider	AM
	The anesthesia provider is responsible for the transport of the patient to a suitable postanesthesia recovery area and the detailed transfer of care to an appropriately trained healthcare worker	PM
**Preanesthetic Assessment and Consent**	The patient must be assessed by the anesthesia provider prior to administration of anesthesia, preferably prior to entry into the operating room, and an appropriate anesthetic plan formulated and documented in the patient’s medical record	AM
	Consent consistent with hospital policy, preferably written, should be obtained	AM
**Preanesthetic Checks**	The anesthesia provider must ensure that the facilities and personnel are adequate for the delivery of safe anesthesia and all medications and equipment (including the anesthesia machine delivery system) have been checked prior to commencing the anesthetic	AM
**WHO Safe Surgery Checklist**	The use of the checklist, appropriately modified for local conditions and priorities	NM
**Record Keeping**	A record of the details of each anesthetic should be made and preserved with the patient’s medical record	AM
**Postanesthesia Care**	All patients who have had an anesthetic (general anesthesia, moderate or deep sedation, regional anesthesia) should remain where anesthetized until recovered or be transported safely to a specifically designated recovery area for postanesthesia recovery	AM
	The postanesthesia recovery area must be adequately staffed by healthcare workers trained to manage patients recovering from anesthesia and surgery	AM
	Oxygen, suction, a means of ventilation (eg, self-inflating bag-mask system), and emergency resuscitation medications must be immediately available	PM
**Transfer of Care and Delegation of Care**	When responsibility for care is transferred from one anesthesia provider to another, or to a nurse or other healthcare worker, all relevant information about the patient’s history, medical condition, anesthetic status, and plan should be communicated to that person	NM
**Pain Management**	All patients are entitled to appropriate efforts to prevent and alleviate postoperative pain using appropriate medications and modalities	PM

Abbreviations: *AM* always met, *PM* partially met, *NM* never met

### Anesthesia provider’s suggestions for safe anesthesia

Anesthesia providers were asked to make free-text comments about ways in which anesthesia safety could be improved in their hospital (Table 8). The main categories were improvements in equipment, availability of anesthetic drugs, access to reliable monitoring, and more training opportunities. These comments indicate that the anesthesia services had long been limited by the economic conditions of the region and that providers had difficulties in maintaining the safety of anesthesia with limited medical expense.

**Table 8:** Anesthetists’ suggestions and comments for making anesthesia safer^a^

Suggestions	n
Better availability of anesthetic equipment eg. anesthesia machine	15
Better availability of monitors, eg. carbon dioxide detector	15
Better salary	14
Better availability of resuscitative drugs and equipments	12
Timely repair of the equipment	10
Better availability of medical supplies eg. electrode patch for ECG	10
Theoretical and practical training	8
Comments	
‘The two anesthesia machines in the operating room are used for a longtime, lack of maintenance and have serious air leak problem.’	
‘We added isoflurane in the only sevoflurane vaporizer, which makes it hard to control the depth of anesthesia.’	
‘With the help of the Internet, we are keen to learn the latest advances in anesthesiology, but because of the lack of equipment and training, our ability to progress was limited.’	
‘One of the serious problems I have encountered is intraoperative awareness. This is a problem that may cause great trauma to patients. It is also a legal issue, but we have ignored it.’	
‘In order to control medical expenses, we used the least amount of drugs and materials, which damages the safety of anesthesia.’	
‘The process we use to dispense large bottles of medication to each patient can lead to contamination.’	

^a^Suggested by the anesthesia providers from the questionnaire

Data are presented as the number of staff members who made suggestions; the total number of staff surveyed was 15.

## Discussion

As the rate-limiting step for provision of safe anesthesia, the quality of anesthesia service is not only related to the outcomes of surgical care but is also an important indicator of the level of medical development [17–22]. In this study, we described the anesthesia service in a main public hospital in Phnom Penh for the first time and identified the problems of anesthesia safety according to the ISSPA. Through questionnaires and interviews with staff in the anesthesiology department, we proposed suggestions for further improvement of the safety of anesthesia that are worthy of reference by future medical assistance teams.

In this survey, we found that the proportion of patients who underwent general anesthesia was 54.3%, which is higher than that in other developing countries [23]. One reason for this high proportion is that the tools and materials for epidural anesthesia were unavailable, and a large number of abdominal operations were performed under general anesthesia. This finding also indicates that with continuous medical assistance and technical support, the drugs and instruments required for general anesthesia are basically guaranteed, and general anesthesia is becoming a routine anesthesia service. However, we also found that the concept of balanced anesthesia had not been implemented, and no regional block with sedative or analgesic measures was performed. This may have been due to the difficulty in obtaining drugs such as dexmedetomidine or midazolam. However, it also indicates that the anesthesia service provider was limited in his or her ability to implement the concept of comfortable anesthesia on the basis of ensuring the safety of anesthesia.

As the population of the world rapidly ages, the amount of surgery being performed in older patients is also increasing [24]. In this study, patients aged > 60 years accounted for 20.9% of the entire sample population, which is high for a country in which the estimated life expectancy is around 70 years [25]. Still, age-related diseases such as ischemic heart disease, stroke, and lower respiratory tract infections are the main causes of death in Cambodia [26]. Under the limited conditions of this country, the aging population poses a great challenge to anesthesia safety. First, there were difficulties in preoperative evaluation. In this survey, we often encountered missing and inadequate medical records and difficulty in acquiring patients’ medical history. Even when the disease had been clearly diagnosed, the treatment was often unstandardized. Second, to balance the medical expenses, doctors often had to omit some examinations such as 24-h dynamic ECG, pulmonary function tests, or coronary angiography tests; thus, some occult diseases could not be diagnosed before surgery. Third, inadequate intraoperative monitoring and poor control of the intraoperative anesthesia depth, which relied solely on oxygen saturation and noninvasive blood pressure monitoring, prevented the detection of problems during the operation in a timely and effective manner and thus increased the risk of perioperative complications in patients of advanced age.

The factors that affect the safety of anesthesia are ultimately associated with Cambodia’s economic development. Because of limited resources, early medical assistants generally performed general anesthesia under the monitoring of oxygen saturation [7]. This method may have resulted in the formation of habits among local anesthesiologists through years of inheritance. Because most of the surgeries in our survey were minor surgeries, a simple pulse oximeter can monitor the blood oxygen saturation of patients during surgery. However, in an emergency such as an allergic reaction, pulse oximetry may miss the best time for rescue, resulting in serious consequences such as death. Our study emphasizes the importance of ensuring the availability of basic monitoring and rescue equipment to improve the level of anesthesia safety. Acquisition of an ECG monitor, end-tidal carbon dioxide monitor, blood gas analyzer, and defibrillator in the operating room can greatly increase the safety of anesthesia. Based on the rational allocation of basic equipment and drugs, a regional anesthesia safety management team should then be established to conduct anesthesia quality control. Reporting of severe adverse events, regular anesthesia safety quality inspections, and personnel training are important measures to effectively improve anesthesia safety [26].

Many medical teams have recently been sent to Cambodia for medical assistance services, which to some extent have alleviated the shortage of medical services in Cambodia [27]. Especially for some surgical services performed on excellent-quality professional medical ships, the overall incidence of anesthesia-related complications and postoperative complications approached the level in developed countries [28]. However, these medical assistance actions have not fundamentally promoted the quality of medical services in developing countries. Donation of medical equipment and medications is another common form of assistance that can quickly improve the treatment capacity of medical institutions. However, there are still problems with maintenance of the equipment [29]. The present investigation revealed that the anesthesia machine in the operating room had air leakage problems that could not be repaired, which poses a great danger to the implementation of general anesthesia. We also found that the basic equipment and medications that affect the safety of anesthesia are still largely missing, necessitating continued medical assistance. Finally, in conducting this survey, we fully understood the local anesthesiologists’ desire for knowledge. Young anesthesiologists, who have received a Cambodia medical education based on western education system, with the same training time and courses as western medical education, are especially skilled at using the Internet and are very interested in new techniques such as ultrasound-guided nerve block. However, they tend to pay more attention to advanced anesthesia technologies than the progress in anesthesia guidelines, and it is worthy of the attention of future medical assistance teams.

This study has several limitations. First, the hospital we surveyed is a large public hospital; we did not collect data from private hospitals. According to the records from the Cambodian Society of Anesthesia Critical Care and Emergency Medicine [30], anesthesiologists at Preah Ket Mealea Hospital tend to have better education and training experience than anesthesiologists in other hospitals, and the patient population they serve is larger than that of other hospitals. Second, we should also recognize that Cambodia still faces a critical shortage of medical resources and has a huge gap between urban and rural medical services [31]; therefore, the results of this study can only reflect the level of anesthesia services in the cities. Third, the sample size was too small to make firm conclusions on the safety of anesthesia practice for rare events such as death [4]. Because of missing data records, the incidence of anesthesia-related complications was obtained through questionnaires rather than from records. Still, as the operating rooms in Preah Ket Mealea Hospital did not perform obstetric and pediatric surgery, our results did not include data on obstetric and pediatric patients. A comprehensive and well-designed further prospective observational study should be done to estimate the incidence of anesthesia-related and postoperative complications [32].

## Conclusion

In this study, we have herein described the status of the anesthesia service carried out in one of the largest public hospitals in Phnom Phen. According to the ISSPA-recommended standards, the most significant risk factors regarding anesthesia safety are the insufficient training of anesthesiologists, the lack of monitoring equipment, and the lack of emergency medications. We recommend that all medical institutions in low-income countries improve their anesthesia services in accordance with the ISSPA so that all patients in each country can have access to safe anesthesia.

## Supplementary information

**Additional file 1: Supplemental Table 1.** Adverse event definitions and **Supplemental Table 2**. Comparison of economic, health care, and anesthesia workforce in the United Kingdom and Combodia.

## Data Availability

The datasets generated and analyzed during the current study are available from the corresponding author on reasonable request.

## Abbreviations

ASA: American Society of Anesthesiologists
CI: Confidence interval
ECG: Electrocardiogram
ISSPA: The International Standards for a Safe Practice of Anesthesia
WHO: World Health Organization
PPP: Purchasing power parity
