# Supplementary material for: The challenge of safe anesthesia in developing countries: defining the problems in a medical center in Cambodia
Source: BMC Health Serv Res. 2020 Mar 12;20:204. doi: 10.1186/s12913-020-5068-z (PMC7068932; doi:10.1186/s12913-020-5068-z)
Supplement: Supplementary file 1 — Additional file 1: Supplemental Table 1. Adverse event definitions and Supplemental Table 2. Comparison of economic, health care, and anesthesia workforce in the United Kingdom and Combodia. [file 12913_2020_5068_MOESM1_ESM.doc]

**Supplemental Table 1.** Adverse event definitions.

| **AIRWAY/PULMONARY EVENTS** | |
| --- | --- |
| Unanticipated difficult airway | Unplanned inability to ventilate the lungs with or without airway adjuncts and/or inability to intubate the trachea using conventional laryngoscopic technique. |
| Hypoxia | SPO_2_＜95%，if patient is already on supplemental oxygen |
| Laryngospasm | A laryngospasmis a muscle spasm in the vocal cords that can lead to problems with speaking and breathing. |
| Bronchospasm | Bronchospasm is a sudden constriction of the muscles in the walls of the bronchioles, causing restricted airflow. |
| Aspiration | Clinical diagnosis of aspiration with radiologic findings. |
| Pulmonary edema | Pulmonary edema is defined as the abnormal accumulation of fluid in the pulmonaryextravascular space. eg. cardiogenic pulmonary edema |
| Unplanned re-intubation | Patient requires placement of tracheal tube or other airway device within 6 hours of extubation including re-intubation after accidental extubation. |
| **CARDIAC EVENTS** | |
| Perioperative myocardial infarction | Detection of rise and/or fall of cardiac biomarkers ECG changes consistent with MI +/- chest pain if markers unavailable |
| Arrhythmia | New PVCs, bradycardia, atrial fibrillation, or other dysrhythmias req. unanticipated Rx. |
| Hypotension & Hypertension | NIBP＞140 mmHg or ＜90 mmHg |
| Cardiac Arrest | The cessation of cardiac mechanical activity confirmed by the absence of signs of circulation. |
| **NEUROLOGIC/OTHER EVENTS** | |
| New stroke | Central neurologic signs and/or symptoms of acute onset and duration of > 24 hours, of presumed vascular etiology, and not consistent with peripheral nervous injury or disease. |
| Seizure | Seizure should be treat with propofol or diazepam |
| Position injury | Positioning-related injuries include perioperative blindness, venous air embolism, compartment syndrome and surgical positioning-related peripheral nerve injury. |
| Recall | Patient report of specific identifiable intraoperative events or conversations while under general anesthesia, after induction but excluding the emergence process. |
| **REGIONAL EVENTS** |  |
| High Spinal | Paralysis higher than T4 +/- hypotension, bradycardia, or respiratory insufficiency. |
| Failed Regional | Regional anesthesia failed |
| Local anesthesia systemic toxicity | Neurologic or cardiovascular signs and/or symptoms attributed to local anesthetic injection occurring within one hour of injection. |
| Peripheral neurologic deficit | Clinical diagnosis of residual sensory and/or motor and/or autonomic block 72 hrs. after local anesthetic, without identifiable etiology when no regional anesthetic related infection present. |
| **MEDICATION/TRANSFUSION EVENTS** |  |
| Anaphylaxis | Clinical diagnosis of a severe, life-threatening allergic response. |
| Malignant hypothermia | Clinical diagnosis of suspected MH during or after exposure to anesthetics agents and/or use of dantrolene |
| Transfusion reaction | Hemolytic reaction involving administration of blood or blood products. |
| Prolonged PACU stay | Patient in PACU > 4 hours |
| Perioperative shivering | Shivering due to the combination of anesthetic-induced thermoregulatory impairment and exposure to a cool environment |

**Supplemental Table 2.**  Comparison of economic, health care, and anesthesia workforce in the United Kingdom and Cambodia

|  | Cambodia | United Kingdom |
| --- | --- | --- |
| Total population (thousands) | 15,762 | 65,789 |
| Gross national income per capita (PPP, International $) | 2890 | 35760 |
| Life expectancy at birth male/female (years) | 67/71 | 80/83 |
| Total expenditure on health per capita (International $) | 183 | 3377 |
| Physician anaesthesia providers; n | 450 | 11549 |
| Physician anaesthesia providers per 100,000 population | 2.89 | 17.85 |
| Nurse anaesthesia providers; n | 100 | 0 |
| Percentage of physician providers that have an anaesthetic qualification | 33.3% | 59.3% |
| Minimum duration of training (years) for physician anaesthesia providers | 3 | 5 |
| Typical duration of training (years) for nurse anaesthesia providers | 2 | 0 |

Abbreviations: PPP, purchasing power parity. Sources: https://www.wfsahq.org/ and http://www.who.int.
